# Insights into the physical and chemical properties of struvite crystal surfaces in terms of the effectiveness of bacterial adhesion

**DOI:** 10.1038/s41598-023-32758-3

**Published:** 2023-04-05

**Authors:** Jolanta Prywer, Agnieszka Torzewska, Michał Cichomski, Paweł Piotr Michałowski

**Affiliations:** 1 Institute of Physics, Lodz University of Technology, Wólczańska 217/221, 93-005 Łódź, Poland; 2 Department of Biology of Bacteria, Faculty of Biology and Environmental Protection, University of Lodz, Banacha 12/16, 90-237 Łódź, Poland; 3 Department of Materials Technology and Chemistry, Faculty of Chemistry, University of Lodz, Pomorska 163, 90-236 Łódź, Poland; 4 Łukasiewicz Research Network - Institute of Microelectronics and Photonics, Aleja Lotników 32/46, 02-668 Warsaw, Poland

**Keywords:** Biophysical chemistry, Surfaces, interfaces and thin films

## Abstract

In this paper, we present the results of research on the physicochemical properties of two selected faces of the struvite crystal, which is the main component of infectious urinary stones. Two main faces, (001) and ($00\overline{1}$), ending the *c*-axis, were selected for the study. These faces are not related by symmetry relations, which means, among other things, that they should have a different atomic structure, which was confirmed experimentally. In addition, the studies show that the tested surfaces have hydrophilic properties, however, the ($00\overline{1}$) face is more hydrophilic compared to the (001) face. The physicochemical properties of the crystal as a whole, as well as the physicochemical properties of these faces influence the magnitude of adhesion. The adhesive force in both water and artificial urine is greater for face ($00\overline{1}$) compared to face (001). The assessment of the adhesion of *Proteus mirabilis* bacteria in artificial urine also shows that the adhesion is greater for face ($00\overline{1}$) than for face (001). The adhesion of bacteria to the examined faces of the struvite crystal, and in particular the increased adhesion of bacteria to the face ($00\overline{1}$), may be the first stage of biofilm formation, which may result in a high rate of recurrence of infectious urinary stones after treatment.

## Introduction

Struvite (magnesium ammonium phosphate hexahydrate; MgNH_4_PO_4_·6H_2_O) is the main component of infectious urinary stones, accounting for 10%^1^ to 30%^2^ of all human urinary stones. Struvite is formed when the urinary tract becomes colonized by bacteria (such as *Proteus*) that produce an enzyme called urease. Urease catalyses the hydrolysis of urea (NH_2_)_2_CO with the formation of ammonia, NH_3_^3^. As a consequence, the urine pH increases. The increasing urine pH leads to an increased concentration of NH_4_^+^, CO_3_^2−^ and PO_4_^3−^ ions. These ions together with the magnesium ions Mg^2+^ present in urine lead to struvite crystallization. Struvite stones can grow rapidly and, if not properly treated, can turn into a large stone that fills the entire intrarenal collecting system. Patients with infectious urolithiasis who are not treated have an approximately 50% chance of kidney loss^4,5^. A detailed description of the process of the formation of infectious urinary stones along with all specific chemical reactions is given elsewhere^3,6–8^.

The most commonly used treatment for infectious urinary stones is extracorporeal shock wave lithotripsy (ESWL), usually associated with long-term antibiotic therapy. Both of these procedures do not give a fully positive treatment outcome. This is because microorganisms can survive inside the urinary stone^9^ and the ESWL used can release microorganisms into the urinary tract. Such microorganisms can serve as centres of heterogeneous nucleation. Moreover, even dead bacteria can be active centres of recrystallization^9^. Consequently, recurrence after treatment occurs in 50% of cases^10,11^. Another reason for such a high rate of recurrence of infectious urinary stones may be the formation of a bacterial biofilm in which bacteria surrounded by exopolysaccharides are resistant to antibiotics and the host's immune mechanisms. The formation of a biofilm, which is a multicellular community of microorganisms, usually begins with the adhesion of several microorganisms to the surface^12^. Adhesion is largely due to the action of physical forces such as van der Waals forces and electrostatic forces^13,14^. Also, the hydrophobicity and hydrophilicity of a given surface can influence the adhesion to that surface.

The aim of this study is to investigate the physicochemical properties of selected main faces (001) and ($00\overline{1}$) of struvite and to test the effectiveness of adhesion to these faces, in particular bacterial adhesion. Such studies may be all the more interesting as struvite has recently been shown to be a ferroelectric crystal^15^. The polar axis is the *c-*axis, which means that the faces ending this axis, i.e. (001) and ($00\overline{1}$), have a different atomic structure and opposite electric charges. These faces can therefore show different adhesion. These studies are intended to provide an answer to the question of whether the physicochemical properties of the main faces of struvite crystals may affect the adhesion of bacteria to these crystals and, consequently, contribute to the formation of biofilm. This is important because the formation of biofilm may be responsible for the high rate of recurrence of infectious urinary stones after treatment.

## Materials and methods

### Struvite crystals growth from gel medium

**Figure 1:**
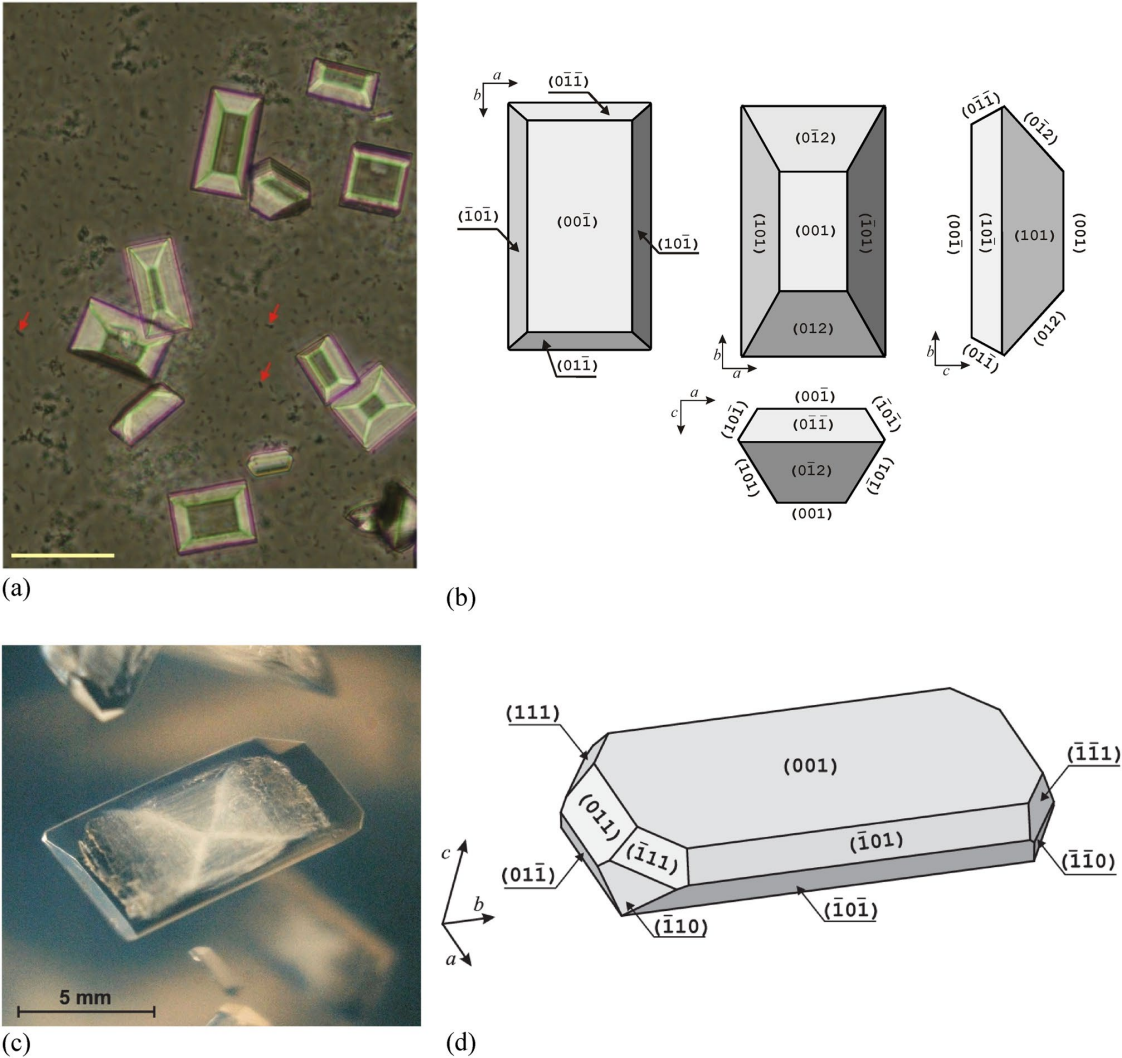
(**a**) Struvite crystals crystallized in artificial urine by the action of bacteria (arrows), scale bar 50 μm; (**b**) a schematic representation of struvite crystal in different observation planes; (**c**) a struvite crystal grown in a gel; and (**d**) a schematic representation of its habit with Miller indices of the individual faces. (**a**) reprinted (adapted) with permission from Ref.^24^. Copyright 2023 American Chemical Society; (**c**) and (**d**) reprinted from Ref.^15^.

In order to study the physicochemical properties of struvite crystalline faces and the phenomenon of adhesion to these faces, struvite crystals in the form of a flat plate of sufficiently large dimensions are needed. Struvite crystals grown in the urine under the influence of bacteria do not meet these criteria. Such crystals are too small for this type of research. Therefore, we have grown crystals in a metasilicate gel environment using a single diffusion gel growth technique. This method is described in many literature sources (e.g. Ref.^16^). The specific growth conditions used in this case are the same as those used in Ref.^15^. One of the obtained struvite crystals is shown in Fig. 1c and d.

### Secondary-ion mass spectrometry (SIMS) technique

Secondary-ion mass spectrometry (SIMS) was used to determine the atomic composition of the two main faces of struvite, namely (001) and (00 $\overline{1 }$). CAMECA SC Ultra instrument was used for the following SIMS measurements and the measurement procedure was designed in such a way that only the crystal surface was tested: samples were bombarded with Cs^+^ primary ions with extremely low impact energy (100 eV) and relatively high beam incident angle (75°). In addition, the ion dosage was limited to about 5 × 10^12^ atoms/cm^2^ and thus less than 1% of the tested surface was damaged during ion bombardment. This means that there was a very small chance that the same spot would be hit twice in one experiment. Due to the very low energy that the ions transmitted to the sample, it could be assumed that the emission of secondary ions occurs only from the very surface of the crystal.

Positive detector polarity was used for magnesium detection whereas negative for phosphorous and oxygen. Due to the presence of residual gases in the analysis chamber, an average oxygen level was determined on a reference sample (FZ silicon) and subtracted from the final result. No background was present for phosphorous and magnesium.

### Wettability and adhesion force measurement

The wettability of the tested surfaces (001) and ($00\overline{1}$) was measured in the laboratory atmosphere at a relative humidity of 45 ± 5% and temperature of 22 ± 2 °C using a DSA-25 Drop Shape Analysis System (KRÜSS GmbH). The deionized water (POCH S.A., Gliwice, Poland) was used for making contact angle measurements. Drops with a volume of 2 μL were deposited on the surfaces with the use of an automatic syringe. The static contact angle values were reported for five distinct drops deposited on each sample.

The work of adhesion ${W}_{\mathrm{S}}$ was calculated based on Eq. (1). This equation introduced a quantity on real surfaces of the apparent contact angle of static water for a water drop of a solid surface using the Young-Dupre equation^17,18^:

1 $$W_{{\text{S}}} = \gamma_{{{\text{LW}}}} \left( {1 + {\text{cos}}\Theta_{{{\text{WCA}}}} } \right)$$

where ${\text{cos}}\Theta_{{{\text{WCA}}}}$ is the equilibrium (Young's) contact angle and $\gamma_{{{\text{LW}}}}$ is water surface tension.

Measurement of the nano-scale adhesion force on the two main faces (001) and ($00\overline{1}$) of the struvite crystal was performed with an atomic force microscope (AFM) in air and in a urine drop. In the case of measurement in air under ambient conditions, the AFM tests were carried out using the Solver P47 apparatus (NT-MDT, Moscow, Russia) Adhesive forces were investigated using a rectangular cantilever made of Si_3_N_4_ with a spring constant *k* = 0.65 ± 0.07 N/m. The radius of the curvature of the tip was less than 20 nm. This method has been described before, for example in Ref.^19^. The adhesive force was obtained from the force–distance curve as a calculation of the pull-off force^20^. Obtained data from different points on a given crystal surface were then averaged from ten force–distance curves.

The adhesion force to the (001) and (00$\overline{1}$) faces of struvite was also assessed with a Bioscope Resolve atomic force microscope (Bruker) in a drop of artificial urine. The artificial urine used for the experiment consists of the following components, the concentrations (g/L) of which are given in brackets: calcium chloride dihydrate CaCl_2_·2H_2_O (0.651), magnesium chloride hexahydrate MgCl_2_·6H_2_O (0.651), sodium chloride NaCl (4.6), sodium sulfate Na_2_SO_4_ (2.3), potassium dihydrogen phosphate KH_2_PO_4_ (2.8), potassium chloride KCl (1.6), ammonium chloride NH_4_Cl (1.0), trisodium citrate Na_3_C_6_H_5_O_7_ (0.65), disodium oxalate Na_2_C_2_O_4_ (0.023), urea CH_4_N_2_O (25.0), and creatinine C_4_H_7_N_3_O (1.1)^21^. This composition reflects components quantitatively and qualitatively equivalent to those present in the urine of a healthy person. In other words, such urine composition reflects the content of mineral components corresponding to the average 24-h concentration in the urine of a healthy person. The artificial urine was prepared by dissolving chemicals (Sigma-Aldrich) of reagent grade purity in distilled water. The solution of urine was then filtered through a membrane filter with a pore size of 0.2 μm. Urine pH for adhesion tests was 6.3. Gel-grown struvite crystals, rinsed and slightly dried before the measurement, were glued to metal discs, which were then placed on a piezoelectric scanner and poured over with a drop of artificial urine. The stabilization of the sample was usually achieved from half an hour to an hour after the sample was placed on the scanner and then the measurement could be started. The measurement was performed at room temperature. The force–displacement curves were recorded with a Nova Scan spherical probe (cantilever spring constant *k* = 0.076 N/m, sphere diameter 5 µm). The probe was made of glass and covered with gold. The cantilevers to which the probes were attached were vibrometrically calibrated by the manufacturer. Before the measurement no-touch calibration was performed (by means of thermal vibrations) to obtain a Deflection Sensitivity (DS) value of 41 nm/V. Measurements of force–displacement curves of the piezoelectric scanner were made for two crystal surfaces: (001) and ($00\overline{1}$). Measurement parameters: curve length (ramp size) = 1 μm; single curve collection frequency (ramp rate) = 0.5 Hz, load = 1nN, number of curves 100.

### Evaluation of bacterial adhesion to the crystal surfaces (001) and ($00\overline{1}$)

The adhesion of bacteria to the surface of gel-grown struvite crystal was tested in conditions resembling the conditions in the urinary tract during bacterial infection. For this purpose, the adhesion experiment was carried out in artificial urine with the use of the bacteria *Proteus mirabilis*, strain C11-K obtained by courtesy of the 2nd Department of Urology, Medical University of Lodz, Poland, and isolated from a kidney stone of a patient of this clinic. Before the experiment bacteria were cultured on a tryptic soy broth (TSB, BTL, Poland) for 18 h at 37 °C and then suspended in artificial urine. Artificial urine was prepared as described in section "Wettability and adhesion force measurement". In addition to the ingredients listed there, one ingredient was added in the presence of bacteria, namely TSB, used to stimulate bacterial growth. Struvite crystals obtained as described in section "Struvite crystals growth from gel medium" were rinsed with sterile water and then transferred to a round bottom tube containing a bacterial suspension (10^7^ CFU/ml) in a volume of 0.5 ml of artificial urine. Samples prepared in this way were incubated for 1 h at 37 °C with gentle stirring (100 rpm; shaker incubator Infors HT). After incubation, the crystals were washed three times with distilled water, transferred to an Eppendorf tube, then 20 µM of SYTO 13 dye aqueous solution of (Ex 488 nm, Em 514 nm, Invitrogen Molecular Probes) was added and samples were stained for 30 min in the dark at room temperature. After rinsing three times with water, the crystals were observed in a Leica SP-8 inverted confocal microscope with excitation/emission peaks of 488/514 nm. The image analysis was performed using LAS AF lite 4.0 image processing software.

## Results

Struvite crystallizes in the orthorhombic noncentrosymmetric polar space group *Pmn*2_1_ (No. 31). The point group is *mm*2, and the unit cell parameters are as follows: *a* = 6.9650(2) Å, *b* = 6.1165(2) Å, and *c* = 11.2056(3) Å^22^. The crystal is polar, the polar axis is the *c-*axis. This means that the faces that terminate the *c-*axis are not related by symmetry. Consequently, the two ends of the *c* direction are not symmetrically related. Thus, the surfaces that terminate the crystal at both ends have different properties and usually grow differently. The habit of struvite crystals characteristic for growth in the urine environment is shown in Fig. 1a. The number of crystalline surfaces present in a crystal may slightly differ from one crystal to another. However, the main and most significant faces are the (001) and ($00\overline{1}$) faces, which are perpendicular to the polar *c-*axis; Fig. 1b. These faces (001) and ($00\overline{1}$) almost always appear (sometimes the face (001) disappears to form an edge—this phenomenon as well as a full analysis of struvite habit and morphology is presented in Ref.^23^). These faces, as the crystal is noncentrosymmetric, are not related by symmetry. It is known from the literature^15^ that the crystal exhibits spontaneous polarization, which means the existence of a net electric dipole along with *c-*axis. Specifically, face (001) has a negative and face ($00\overline{1}$) positive electric charge.

**Figure 2:**
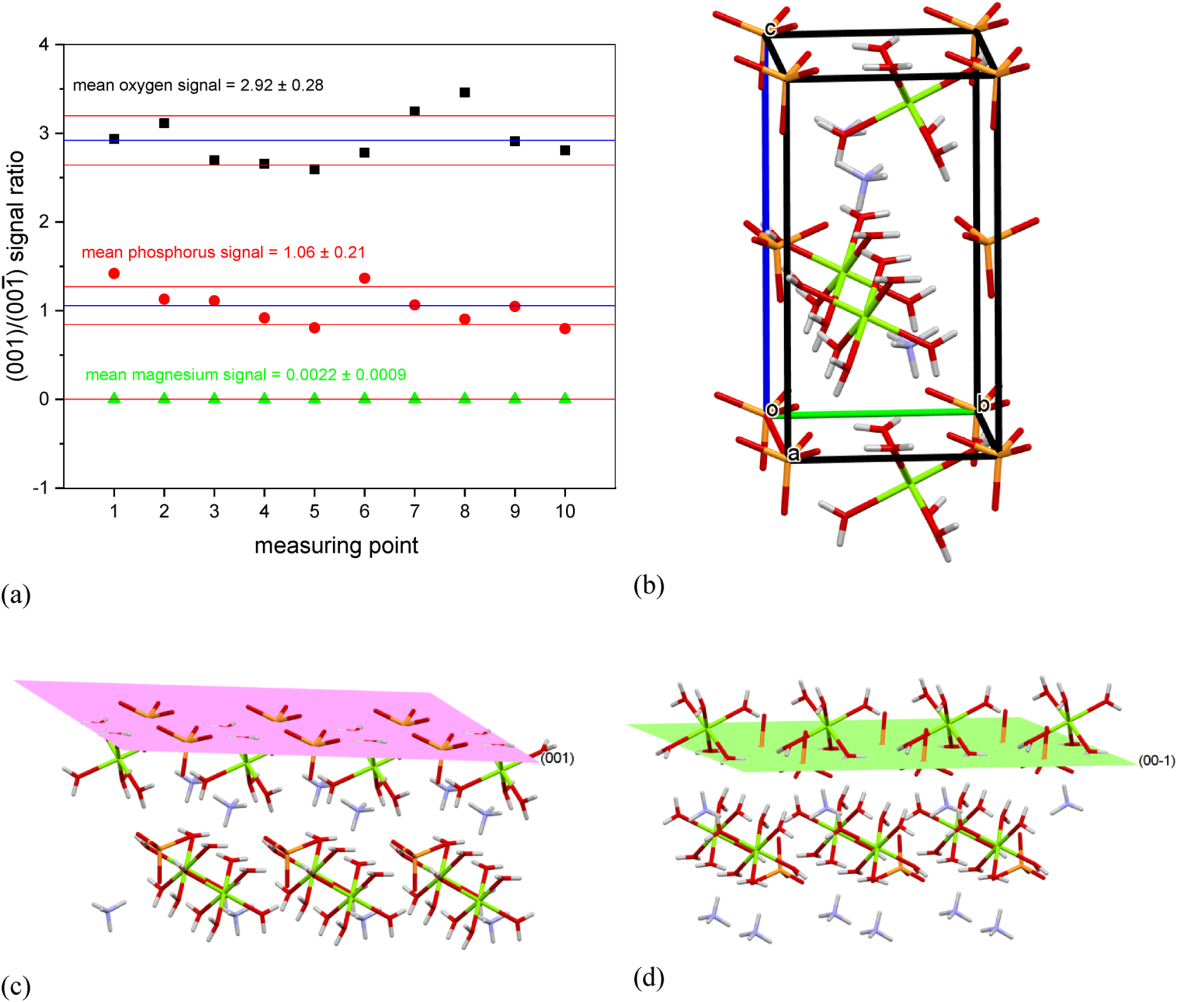
(**a**) ratio of the intensities of the measured signals for oxygen, phosphorus, and magnesium for surface (001) relative to the surface (00$\overline{1 }$). Measurements obtained using the SIMS technique. (**b**) Arrangement of atoms in the struvite crystal unit cell. The crystal is built from PO_4_^3-^, NH_4_^+^ and Mg[H_2_O]_6_^2+^; *c*-axis is polar. The atomic structure of surfaces (**c**) (001) and (d) (00$\overline{1 }$). (**b**), (**c**) and (**d**): O, H, Mg, P and N atoms are colour coded respectively: red, whitish gray, green, orange, and purple.

The different charges on these faces suggest that these faces have a different atomic structure. In order to check it, the first step is to grow a crystal of sufficiently large size, adequate to the measurement techniques used. Therefore, the crystals were grown in metasilicate gel using a single diffusion gel growth technique as described in section "Struvite crystals growth from gel medium". An exemplary crystal obtained by this method is shown in Fig. 1c and d. The face ($00\overline{1}$) is the bottom face (not visible in Fig. 1d) parallel to the top face (001). The crystal grown in this way, and of such a habit, was used to study the atomic structure of both faces using the SIMS technique. In particular, we focused on measuring the amount of oxygen, phosphorus, and magnesium atoms on both faces. The results are shown in Fig. 2. As can be seen from Fig. 2, the measurements were made ten times for ten different points on a given crystalline face. The mean ratio of the intensities of the measured oxygen signals for surface (001) relative to the surface ($00\overline{1}$) is 2.92 ± 0.28, which means that the face (001) has three times more oxygen atoms than the face ($00\overline{1}$). The mean ratio of the intensities of the measured phosphorus signals for surfaces (001) and ($00\overline{1}$) is 1.06 ± 0.21, which means that there are practically the same number of phosphorus atoms on both faces. The mean number of counts of magnesium atoms for the face ($00\overline{1}$) is 22,877.7, while for the face (001) it is only 49.3. This means that the magnesium atoms are only on the face ($00\overline{1}$) and the mean ratio of the intensity of the measured magnesium signals (001)/($00\overline{1}$) is 0.0022 ± 0.0009. This result confirms that the established SIMS measurement procedure is extremely surface sensitive.

The results obtained with the SIMS technique agree well with the atomic structure of (001) and ($00\overline{1}$) faces calculated with Mercury software (Mercury 2020.2.0)^25–28^. Figure 2b shows the distribution of atoms in a unit cell, and Fig. 2c and d of the atomic structure of the faces (001) and ($00\overline{1}$). It is clearly seen that three oxygen atoms protrude from the face (001) from each PO_4_^3−^ anion, and from the opposite face ($00\overline{1}$) only one oxygen protrudes. On the face (001) we also see slightly protruding oxygen and hydrogen atoms derived from H_2_O molecules bound to Mg into the Mg[H_2_O]_6_^2+^ cation. On the face ($00\overline{1}$) we can see whole water molecules bound to Mg into the Mg[H_2_O]_6_^2+^ cation. However, it should be noted that the Mg-bound water molecules were evaporated in the measuring chamber. Therefore, in Fig. 2a, only oxygen atoms bound in the PO_4_^3−^ anion are disclosed. And there are three times more of these atoms on the face (001) compared to the face ($00\overline{1}$). From Fig. 2c and d, we can also conclude that there are as many phosphorus atoms on the face (001) as there are on the face ($00\overline{1}$). These atoms are derived from the PO_4_^3−^ anion. Regarding magnesium, it can be seen (Fig. 2c and d) that there is no magnesium present in the face (001). Magnesium is under the surface of this face. In contrast, magnesium protrudes from the face ($00\overline{1}$) and therefore the magnesium signal was recorded only on the face ($00\overline{1}$); Fig. 2a.

Since the atomic structure of these faces is different and also the electric charge is different, positive on the surface ($00\overline{1}$) and negative on the surface (001)^15^, it should be assumed that these faces will have different effects with the impurities, and the adhesion to both surfaces may also be different. The physical and chemical properties of the surface affect the adhesion efficiency.

**Figure 3:**
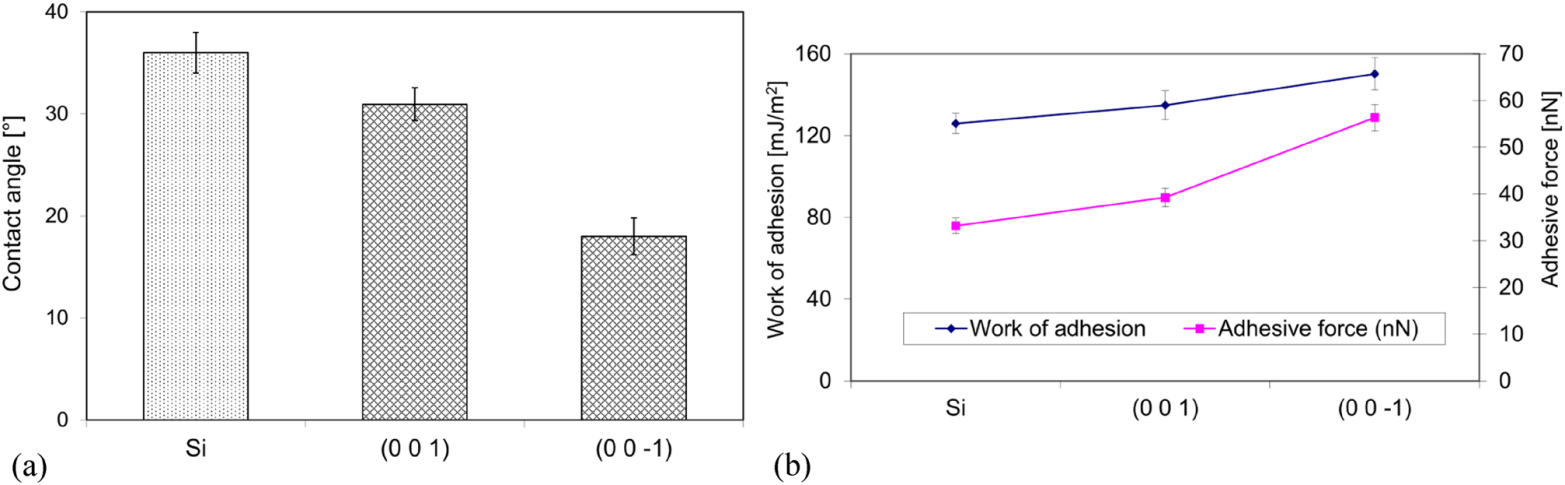
Contact angle (**a**) and (**b**) work of adhesion vs. adhesive force for (001) and (00$\overline{1 }$) faces of struvite.

The parameter influencing the adhesion is the wettability and the work of adhesion ${W}_{\mathrm{S}}$. The wettability, which is represented by the contact angle and the work of adhesion ${W}_{\mathrm{S}}$, can be calculated (Eq. 1) from the measured surface tension. Figure 3 shows the results of the water drop contact angle measurements for the faces (001) and ($00\overline{1}$) of the struvite crystal in relation to the reference surface Si. From this figure (Fig. 3a), it can be seen that the contact angles for water for both faces (001) and ($00\overline{1}$) are less than 90° and are 30.96° ± 1.6° and 18° ± 1.8° respectively. Hence the conclusion that the ($00\overline{1}$) surface has a more hydrophilic character. Figure 3b illustrates the obtained values of the work of adhesion for water and the adhesive force. From this graph (Fig. 3b) it can be seen that both the work of adhesion and adhesive force are higher for the face ($00\overline{1}$) compared to the face (001). Specifically, the work of adhesion is 134.68 mJ/m^2^ and 150.23 mJ/m^2^ for faces (001) and ($00\overline{1}$) respectively, and the adhesive force is 39.24 nN and 56.33 nN, respectively. The results therefore clearly show that in the case of water the adhesion is higher for the face ($00\overline{1}$) than for the face (001). This result is compatible with the measured contact angle values because stronger adhesion results in a greater degree of wetting, which means a lower value of the contact angle.

**Figure 4:**
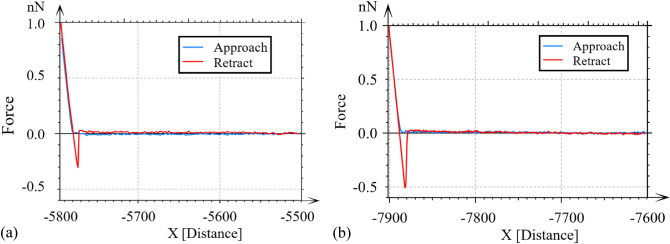
Examples of force-distance curves for struvite faces (**a**) (001) and (**b**) (00$\overline{1 }$) in artificial urine. For the presented curves, the values of the adhesive force for faces (001) and (00$\overline{1 }$) are equal to: 0.30 nN and 0.51 nN, respectively.

For additional verification of the obtained results, the adhesion force was measured with an AFM in a drop of urine (details of measurement in section "Wettability and adhesion force measurement"). Measurements of force–displacement curves of the piezoelectric scanner were made for the bottom ($00\overline{1}$) and top (001) surfaces of struvite. The obtained results indicate that for surface (001), the values of the adhesion force ranged from 0.20 to 0.30 nN, and for surface ($00\overline{1}$) from 0.51 to 0.76 nN. Exemplary force–displacement curves for the crystal top (001) and bottom ($00\overline{1}$) surfaces are shown in Fig. 4.

It should be noted that the obtained values of the adhesion force are very small and for individual faces they fall within a fairly broad range of values. The obtained low adhesion values are related to the parameters of the measuring probe used for the tests (work in the low force regime). Additionally, it should be noted that the stabilization of the sample was usually achieved from half an hour to an hour after placing the sample on the scanner and then the measurement could be started. In addition, we tried to measure several dozen curves, which also took time. With the passage of time, a small drop of urine most likely was evaporating and crystallization of urine components took place, and these crystallized components were adsorbed to the measuring probe. Most likely, this effect is related to the fact that the values of the adhesion force are in quite large ranges. However, irrespective of the absolute values of the adhesion forces obtained, the measurement results show that the adhesion force is greater on the bottom surface ($00\overline{1}$) compared to the top surface (001), which confirms our results obtained with other methods.

**Figure 5:**
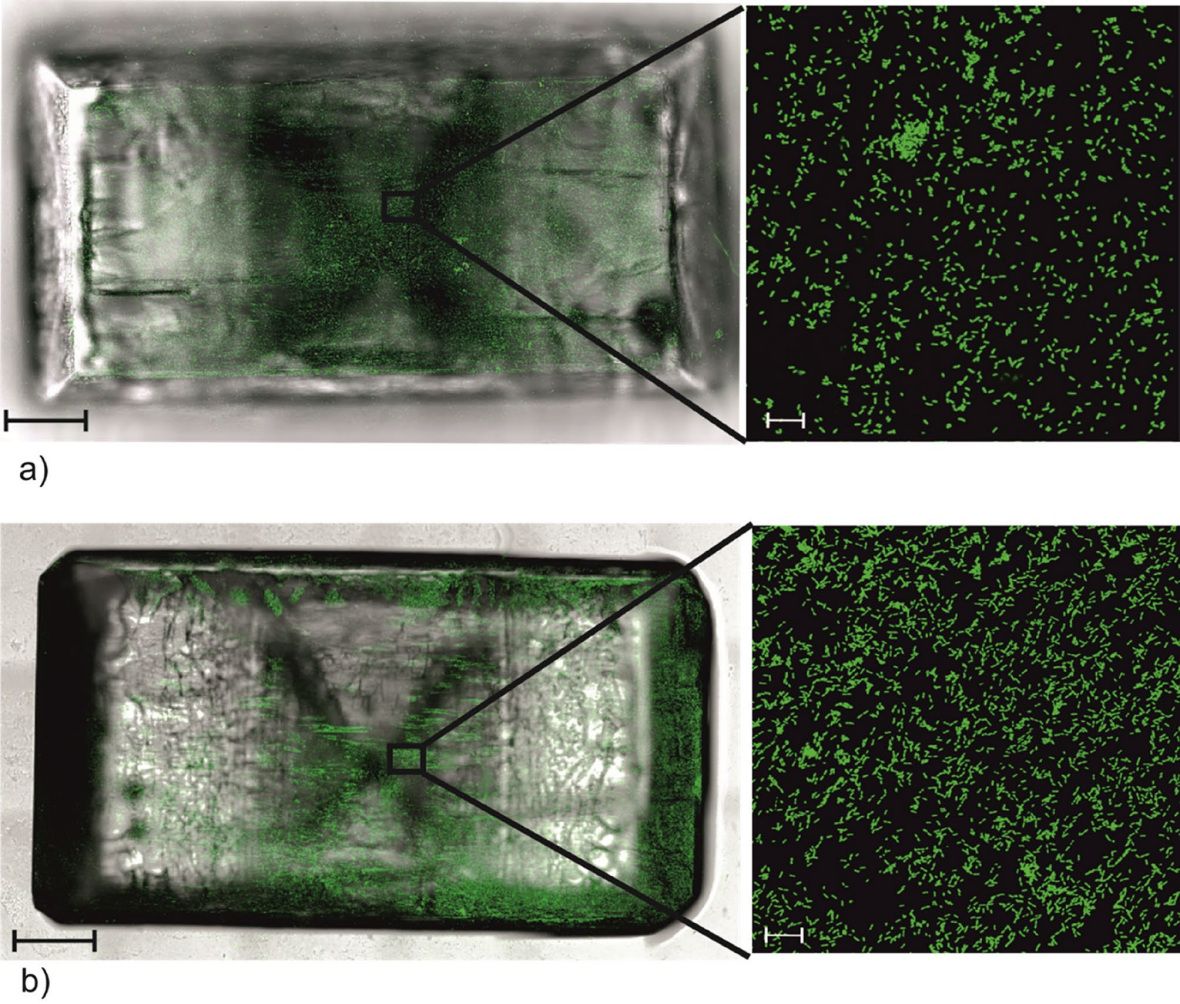
Confocal microscopic observation of *Proteus mirabilis* adhesion to the crystal surface after Syto-13 staining. (**a**) top face (001); (**b**) bottom face (00$\overline{1 }$). On the left, a view of the entire face—scale bar 500 µm (a fluorescence image superimposed with a transmitted light image), on the right, a selected part of the surface, scale bar 20 µm.

In order to verify the measurements of the adhesion forces for the (001) and ($00\overline{1}$) faces of the struvite crystal, we determined the degree of adhesion of *Proteus mirabilis* on these two faces. This species of bacteria was selected for research because these bacteria are most often isolated from infectious urinary stones. The ability of bacterial cells to adhere to the crystal surface was analysed by incubating the crystals in a suspension of *P. mirabilis* in artificial urine. Their presence on the crystal was revealed after staining with the fluorescent dye Syto 13, which specifically stains bacteria by binding to their nucleic acids. The experiment was performed on 6 crystals, Fig. 5 shows a view representative of these results. Green-coloured bacteria are visible on both the bottom and top faces. However, there are more bacterial cells on the bottom face ($00\overline{1}$), which indicates that bacteria have a greater tendency to colonize this surface.

The experiment carried out with *P. mirabilis* in the context of adhesion indicates that the bacteria colonize the ($00\overline{1}$) face to a greater extent than the face (001). The question arises why this is happening. To answer this question, one need to know the zeta potential of *P. mirabilis* in urine. Such research is reported in Ref.^29^. These investigations were carried out in urine of the same composition as in the studies presented in this paper, at the temperature of 37 °C and in the pH range from 6.5 to 9.5 in 0.5 increments. This pH range reflects a change from the normal pH of a healthy human to a pH value corresponding to in vitro infection of urine with urease-positive bacteria. Under such conditions, *P. mirabilis* show a negative zeta potential of approximately − 30 mV in urine, regardless of pH^29^. This means that bacteria can colonize the ($00\overline{1}$) face to a greater extent than the (001) face precisely because they have a negative zeta potential, which means that the bacteria have a negative electric charge on the electric double layer and can be electrostatically attracted to the face ($00\overline{1}$) which is charged positively.

**Figure 6:**
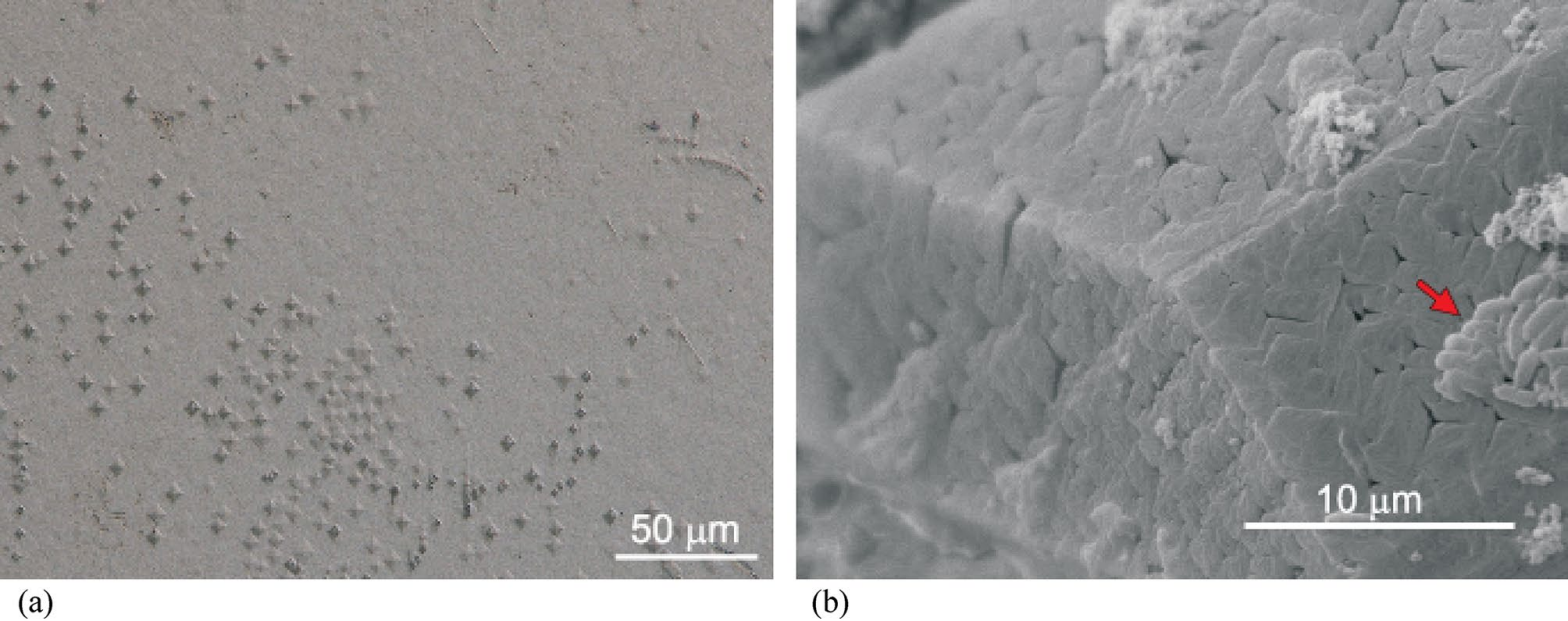
(**a**) Structure of the face (001) of the struvite crystal growing in the gel; etch pits are visible (Keyence VHX-7000 digital microscope). (**b**) Micrograph (JEOL JSM-6610LV scanning electron microscope) of a struvite crystal induced to grow by bacteria in artificial urine with visible bacteria (arrow) on the face (001). The crystal shows a surface texture characteristic of crystallization in the presence of bacteria.

It should be remembered that the presented research was carried out on crystals that grew in a gel, and not in the environment of artificial urine under the influence of bacteria. As it is evident from the studies presented in the literature^30–32^, crystals induced to grow by bacteria usually have a very characteristic surface structure (texture) which is not observed in the case of crystals growing without the presence of bacteria. Figure 6a shows the surface structure of the face (001) of the crystal growing in the gel, and Fig. 6b of the crystal induced to grow by bacteria in artificial urine. A face with visible bacteria (arrow) is the face (001). The structure of the (001) surface in Fig. 6a is smooth compared to that of the crystal induced to grow by bacteria; Fig. 6b. However, in Fig. 6a, the etch pits are also visible. They were most likely formed when the crystal was rinsed in water after removing it from the gel. Typically, etch pits occur at the surface intersection of dislocations. As shown in Fig. 6b, the surface structure of the crystals induced to grow by bacteria is different. The observed surface pattern with such a structuration is characteristic for the presence of bacteria^30–32^. It should be remembered that on crystals induced to grow by bacteria, the adhesion should be greater than with a smooth surface, as the surface structure increases the total surface area. In addition, if the size of the surface irregularities is of the order of the size of the bacteria, this should promote bacterial adhesion since the bacteria can then maximize the contact area with the surface. It should be noted that the surface roughness of struvite crystal faces is on a micrometre scale, which should favour bacterial adhesion. For example, in Ref.^12^, the authors prove that bacteria adhere more to rough surfaces on the micrometre scale than on the nanometre scale.

## Conclusions

The conducted studies of the surfaces (001) and ($00\overline{1}$) of the struvite crystal show that both of these faces show hydrophilic properties, but the face ($00\overline{1}$) is more hydrophilic than the face (001). At the same time, face ($00\overline{1}$) shows greater adhesion force both in the air and in a drop of urine. It seems that the different adhesion force on both these surfaces is related to their different atomic composition as well as a different electric charge resulting from the existence of spontaneous polarization in this crystal. Face (001) is negatively charged, and face ($00\overline{1}$) positively^15^. The experiment carried out with *P. mirabilis* in the context of adhesion indicates that the bacteria colonize the ($00\overline{1}$) face to a greater extent than the face (001). This may be due to the fact that these bacteria show a negative zeta potential of approximately − 30 mV in urine, regardless of pH^29^. This means that these bacteria have a negative electric charge on the electric double layer and can be electrostatically attracted to the face ($00\overline{1}$) which is charged positively. That is, in other words, the physicochemical features of the struvite surface have an influence on the adhesion force. In further studies, it should be checked whether the adhesion of bacteria to the faces of the struvite crystal may be the first stage of biofilm formation. Investigating this problem can give an answer to whether adhesion and possible biofilm formation could contribute to the high rate of recurrence of infectious urinary stones after treatment. In future studies, it may also be considered to take into account other factors, such as the surface energy of struvite faces, which, as indicated by the authors of Ref.^33^, may affect the magnitude of adhesion.

## Data Availability

All data generated or analysed during this study are included in this published article.
